# Prevalence of the HLA-B*5701 Allele and Abacavir Hypersensitivity in Saudi HIV Patients: A Multicenter Study

**DOI:** 10.7759/cureus.48229

**Published:** 2023-11-03

**Authors:** Ali Alsaeed, Zahra Alkhadrawi, Batool Alsadah, Zakia Almudhry, Hawra AlBayat, Fadel Alhadad, Albaraa Dahlawi, Batool Abu Ali, Badr Al muhainy, Taher A Alhaddad, Mousa J Alhaddad

**Affiliations:** 1 Internal Medicine, Dammam Medical Complex, Dammam, SAU; 2 Infectious Diseases, King Saud Medical City, Riyadh, SAU; 3 Internal Medicine, Qatif Central Hospital, Qatif, SAU; 4 Internal Medicine, King Faisal Hospital, Makkah, SAU; 5 Infectious Diseases, King Fahad Hospital, Hofuf, SAU; 6 Internal Medicine, King Fahad Hospital, Hofuf, SAU

**Keywords:** abacavir hypersensitivity, saudi arabia, abacavir, hla-b*5701, hiv

## Abstract

Introduction

Human immunodeficiency virus (HIV) incidence and prevalence are increasing in Saudi Arabia, with a total prevalence of 12,000 in 2020. Treatment of HIV patients includes multiple regimens that may involve abacavir (ABC), which is a potent drug for treating HIV and can be used as a single or combined pill. Unfortunately, its use was limited by the known associated hypersensitivity reaction (HSR). A worldwide literature review over the past decades reported that the incidence of ABC-related HSR is 5-8%.

Methods

The study was a cross-sectional multicentric study involving five governmental hospitals in Saudi Arabia and included all HIV patients who were following in these centers.

Results

Out of 3082 patients, 1293 were tested for HLA-B*5701. The prevalence for ABC-HSR is 1.59%, with variability among the five hospitals, with the highest in King Fahad Hospital in Hafuf (KFH-H) at 4.00% and the lowest in Dammam Medical Complex (DMC) at 0.49%. In previous studies, HLA-B*5701 associated with ABC-HSR varied among different ethnic groups. Our study showed that two patients developed ABC-HSR clinically while they were both negative for HLA-B*5701.

Conclusion

The fact that patients with negative genetic testing are still at risk of developing ABC-HSR makes continuing screening for HLA-B*5701 status essential, as the consequences of missing such a life-threatening HSR could be detrimental.

## Introduction

According to WHO (World Health Organization), the prevalence of human immunodeficiency virus (HIV) in Saudi Arabia was 12,000 cases in 2020, with an incidence of 1000 new cases between 2000 and 2015, with an adult prevalence rate of <0.1% [1-4]. According to the Saudi National AIDS Program (NAP), statistics reported in 2014 showed that the prevalence of patients living with HIV (PLHIV) who were on antiretroviral therapy (ART) was the highest in Jeddah, directly followed by Riyadh, Jizan, Dammam, and western provinces, respectively. About 70.9% of the patients in the kingdom were non-Saudi nationals, while the percentage of Saudis was only 29.1% [2].

Abacavir (ABC) - nucleoside analog reverse transcriptase inhibitors (NRTIs) - which is an anti-HIV used either as a single agent or in combined pills, is well-known for its potency and excellent oral bioavailability [3,4]. Nevertheless, its use is limited due to associated hypersensitivity reactions (HSR).

Reviews from multiple clinical trials over the past decades had consistently reported an incidence of 5-8% for a clinically suspected ABC-HSR [5,6].

The diagnosis requires the presence of two or more of the following symptoms: fever, rash, respiratory, gastrointestinal, and constitutional symptoms, in addition to meeting a predefined exclusion criterion. Life-threatening symptoms are rare; the estimated mortality rate is three per 10,000 patients who received ABC; hence, rechallenging with an ABC or ABC-containing regimen is contraindicated [5,7].

The link between HLA-b*5701 and ABC-HSR in HIV patients was first described in 2002 by Mallal et al. among the Western Australian population. Since then, the patients with positive HLA-b*5701 allele were identified to carry the most significant risk factor for developing ABC-HSR [6,8].

In 2014, the prevalence of HLA-B*5701 carriages among different ethnicities was reported as follows: in Europe (6.8 %), South America (2.6 %), Africa (1%), Mexico (2.2 %), Asia (1.6 %), southeast Asia (11%), and the middle east (2.5%) [9].

Multiple cohorts have demonstrated a significant reduction in ABC-HSR with prospective HLA-B*5701 screening. Initially, these results were mainly among the white population, but further trials showed a comparable effect regardless of race [10-12]. The same results were replicated in the PREDICT-1 and North American trials [8,10,13,14]. In addition, a recent meta-analysis reported a lower incidence of ABC-HSR (1.3% or less) since adopting the prospective HLA-B*5701 screening program by the HIV guidelines in 2008 [7].

This study aims to assess the prevalence of the HLA-B*5701 allele among Saudi HIV patients and its association with an HSR.

## Materials and methods

Study design

This study utilized a cross-sectional multicentric design to investigate the prevalence of the HLA-B*5701 allele among HIV patients in Saudi Arabia. The study was conducted at five governmental hospitals, namely Dammam Medical Complex (DMC) in Dammam, Qatif Central Hospital (QCH) in Qatif, King Fahad Hospital (KFH-H) in Hofuf, King Saud Medical City (KSMC) in Riyadh, and King Faisal Hospital (KFH-M) in Makkah.

Ethical considerations

The study protocol was approved by the Institutional Review Boards (IRBs) of DMC (IM-13, June 12, 2023) and KFH-H (06-E-2023, June 15, 2023). Ethical guidelines were followed throughout the study, ensuring patient privacy, confidentiality, and informed consent.

Study population

The study population consisted of HIV patients who were actively receiving care at the participating hospitals. All HIV patients, regardless of age, gender, or ethnicity, were included in the study to ensure a comprehensive representation of the Saudi HIV population.

Data collection

Data collection was performed by trained healthcare professionals at each participating hospital. Demographic information, medical history, and laboratory results of the HIV patients were collected using standardized data collection forms. All data were anonymized and securely stored to maintain patient confidentiality.

HLA-B*5701 testing

HLA-B*5701 testing was performed using validated laboratory techniques. Blood samples were collected from the HIV patients, and genomic DNA was extracted from the samples. Polymerase chain reaction (PCR) amplification and sequence-specific oligonucleotide probing (SSOP) were employed to detect the presence of the HLA-B*5701 allele. 

Data analysis

Descriptive statistics were used to summarize the demographic and clinical characteristics of the study population. The prevalence of the HLA-B*5701 allele among the tested HIV patients was calculated as the percentage of positive cases. The prevalence and distribution of HLA-B*5701 were analyzed for each participating hospital.

Limitations

It is important to acknowledge certain limitations of this study. First, the sample size may not represent the entire Saudi HIV population. Second, the study focused on HLA-B*5701 testing and its association with HSRs, without considering other genetic markers. Finally, the cross-sectional design limits the ability to establish causality or long-term outcomes.

## Results

A total of 3082 HIV patients were followed across five hospitals in Saudi Arabia and of the 1293 HIV patients tested for HLA-B*5701, 20 (or 1.55%) tested positive. The percentage of HLA-B*5701-positive patients varied across hospitals, with the highest percentage observed at KFH-M (4.00%) in Western Saudi Arabia and the lowest at DMC (0.49%) and Eastern Saudi Arabia. However, all four patients who tested positive for HLA-B*5701 allele in KFH-M were of Myanmar origin (Table 1).

**Table 1:** HLA-B*5701 Testing in HIV Patients in Saudi Arabia DMC, Dammam Medical Complex; KFH-H, King Fahad Hospital-Hofuf; KFH-M, King Faisal Hospital-Makkah; KSMC, King Saud Medical City; QCH, Qatif Central Hospital

Hospital	Total HIV Patients	Tested HIV Patients	HLA-B*5701 Patients	Percentage
DMC	1200	405	2	0.49%
QCH	40	26	1	3.85%
KFH-H	112	62	1	1.61%
KSMC	1600	700	12	1.71%
KFH-M	130	100	4	4.00%
Total	3082	1293	20	1.55%

In the Eastern province, including DMC and QCH, only two cases had clinically diagnosed ABC-HSR, both of which were HLA-B*5701 negative.

Patient A

A 37-year-old Saudi national presented with fever, headache, and photophobia. Investigations confirmed HIV infection with cryptococcal infection. He had a negative HLA-B*5701 test. He was started on induction therapy with amphotericin and fluconazole for two weeks for the treatment of cryptococcal infection, then switched to high-dose fluconazole. According to the patient, he was not taking Bactrim or high-dose fluconazole after discharge (he was non-compliant with therapy and only took one week of therapy after discharge), but he stated he is clearly compliant with the anti-retroviral bill (follow-up CD4 at three months confirmed undetected viral load). Five weeks after discharge, he was started on Triumeq (ABC, lamivudine, and dolutegravir).

Ten days later, he presented to the emergency room with a generalized maculopapular rash associated with severe itchiness. The patient denied fever, gastrointestinal symptoms, or respiratory symptoms. So Triumeq was stopped, and he was switched to descovey and dolutegravir. One week later, he was followed up, and his rash had disappeared.

Patient B

A 32-year-old Saudi national presented to hospital with a history of prolonged fever of six months duration, associated with weight loss and night sweats. He was admitted due to a worsening fever, which occurred daily for two weeks prior to the presentation. He was found to have salmonella bacteremia associated with HIV infection. He completed a two-week course of ceftriaxone after discharge and after receiving a negative HLA-B*5701 result. He was prescribed Triumeq (ABC, lamivudine, and dolutegravir), but took only one tablet. Five minutes later, he complained of severe itchiness and burning sensations all over his body, with palpitations, nausea, headache, and shortness of breath.

Triumeq was stopped, and he was started on descovey and dolutegravir, which he tolerated well without any side effects or other issues. This patient was later found to have a tuberculous iliopsoas abscess and was given anti-tuberculosis therapy. However, the anti-tuberculosis therapy was given two months after this incident. He was not taking Bactrim therapy due to non-availability.

## Discussion

The goal of this study is to look for the prevalence of positive HLA-B*5701 allele in Saudi Arabia. To the best of our knowledge, it is the first study for HLA-B*5701 allele prevalence in Saudi Arabia and Gulf countries.

Among the Saudi population, the percentage of patients carrying the allele was 1.59% (20 patients out of 1259 patients who were tested carried the gene). This prevalence derived from multiple centers around Saudi Arabia, including DMC in the eastern province (0.49%, 371 patients), QCH in the eastern province (3.85%, 26 patients), KFH-H (1.61%, 62 patients), KSMC in Riyadh (1.71%, 700 patients) and KFH-M (4.00%,100 patients).

With a variability of prevalence between hospitals, the highest is in KFS-H at 4.00%, and the lowest is in DMC at 0.49 %, which is considered a relatively low percentage in comparison to other countries.

The reported prevalence of this allele from different parts of the world varied among different ethnicities and races; however, compared to the rest of the world, Saudi Arabia has a lower prevalence of positive HLA-B*5701 allele status, as demonstrated in our results.

The prevalence of this allele was found to be 3% in Iran, 4.98% in Europe (6.49% white and 0.39% black), 4.55% (7.93% white, 0.26% black) in UK, 7.7% in Western Australia (all white), 4% in Portuguese, 6% in Spain, 3.1% in northeastern Brazil, 0.86% in China, 5.6% in Georgia, 1.3% in black Africans, and 0% in Nigeria [11,15-24].

In Saudi Arabia's Eastern province, including DMC and QCH, only two cases were clinically diagnosed (ABC-HSR), both of which were HLA-B*5701 negative. The reaction was in the form of a generalized maculopapular rash associated with severe pruritus, headache, and shortness of breath.

A comparable result was found in a double-blinded clinical trial done in Uganda, where the incidence of clinical ABC-HSR was 2.0% (6/300), and all of the six cases were HLA-B*5701 negative [25].

Unfortunately, Individuals who do not carry the HLA-B*5701 haplotype have the possibility of experiencing an HSR as well. Studies found that combining skin patch tests with HLA testing can help in decreasing rates of false positive reports among HLA-negative individuals [4,24].

Accordingly, genetic screening is considered mandatory before starting ABC treatment. Keep in mind that clinical vigilance remains equally important as genetic testing to aid in detecting the mild form of HSRs, especially in patients with negative genetic testing [7].

Limitations

The study is subject to several limitations, including relatively small sample size, potential regional bias, absence of comparative analysis, incomplete genetic testing, lack of long-term follow-up data, and limited exploration of ethnic diversity within the Saudi population, which should be taken into consideration when interpreting the findings and generalizing them to broader contexts.

## Conclusions

In conclusion, this pioneering multicentric study conducted in Saudi Arabia represents the first investigation in the Arab world to assess the prevalence of the HLA-B*5701 allele among Arab PLHIV. The study's findings have significant implications for personalized medicine and drug safety in this population. The observed prevalence of HLA-B*5701 among Saudi HIV patients, though relatively low at 1.59%, highlights the importance of genetic screening in identifying individuals at risk of HSRs to ABC. The variability in HLA-B*5701 prevalence across different hospitals underscores the need for localized data to inform clinical decision-making. Importantly, the study also identified cases where patients developed ABC HSRs despite testing negative for HLA-B*5701, emphasizing the necessity for continued monitoring and exploring additional genetic markers or risk factors associated with ABC adverse reactions. As the first study of its kind in the Arab world, these findings provide a crucial foundation for further research and contribute to advancing HIV patient care in the region.
